# Effect of *Lactobacillus paracasei* Culture Filtrates and Artichoke Polyphenols on Cytokine Production by Dendritic Cells

**DOI:** 10.3390/nu8100635

**Published:** 2016-10-14

**Authors:** Angelo Sisto, Diomira Luongo, Lucia Treppiccione, Palmira De Bellis, Donato Di Venere, Paola Lavermicocca, Mauro Rossi

**Affiliations:** 1 Institute of Sciences of Food Production (ISPA), National Research Council (CNR), Via Amendola 122/O, Bari 70126, Italy; mirella.debellis@ispa.cnr.it (P.D.B.); donato.divenere@ispa.cnr.it (D.D.V.); paola.lavermicocca@ispa.cnr.it (P.L.); 2 Institute of Food Sciences, National Research Council (CNR), Via Roma 52, Avellino 83100, Italy; mluongo@isa.cnr.it (D.L.); luciatrep@gmail.com (L.T.); mrossi@isa.cnr.it (M.R.)

**Keywords:** probiotic bacteria, immunomodulatory bacterial metabolites, dendritic cells, artichoke phenolic compounds

## Abstract

The most recent trend in research on probiotic bacteria aims at the exploitation of bioactive bacterial compounds that are responsible for health-promoting effects and suitable for medical applications. Therefore, the main purpose of this study was to ascertain if the immunomodulatory effects of *L. paracasei* strains on dendritic cells (DCs) were caused by bacterial metabolites released in the culture medium. For that reason, bacterial strains were grown in two media generally used for the culture of DCs, and the effects of culture filtrates on the maturation of DCs and cytokine production were evaluated. Moreover, to reveal potential synergistic effects on the immunomodulation of DCs, an artichoke phenolic extract (APE) was added to the media before bacterial growth. The experiments pointed out an interesting anti-inflammatory activity of a culture filtrate obtained after growing a probiotic *L. paracasei* strain in one of the media supplemented with APE. Therefore, this culture filtrate—which combines the anti-inflammatory activity and the other well-known health-promoting properties of artichoke phenolic compounds—could represent the basis for future particular exploitations.

## 1. Introduction

Bacterial strains of the species *Lactobacillus paracasei* can be considered as common inhabitants of the human intestinal tract, and they are usually also isolated from foods such as milk, dairy products, and fermented vegetables. This species is generally recognized as safe (GRAS and “Qualified Presumption of Safety”-QPS status) because of a long history of safe human consumption [1,2]. Accordingly, a number of *L. paracasei* strains have been studied to define their strain-specific health-promoting properties and selected to be used as probiotics. In particular, strain *L. paracasei* IMPC 2.1 has been studied and used for the development of an innovative patented functional food [3,4,5,6,7] whose commercialization has been authorized by the Italian Ministry of Health, based on the association of that probiotic strain with vegetables. The probiotic/vegetable joining has the aim of broadening the variety of probiotic food types using a suitable vegetable carrier rich in functional components. In this regard, artichokes contain polyphenols—in particular flavonoids and hydroxycinnamic acid derivatives—which are known for their antioxidant properties. The main phenolic compounds contained in artichoke head are caffeoylquinic acids, above all chlorogenic acid (5-*O*-caffeoylquinic acid) and two dicaffeoylquinic acids (1,5-*O*- and 3,5-*O*-dicaffeoylquinic acid) [8,9]. Therefore, artichoke was selected as a possible vegetable carrier of strain *L. paracasei* IMPC 2.1, whose probiotic aptitude and efficacy were also studied in association with that food matrix. In particular, the probiotic activities of strain IMPC 2.1 (recognized by the European Food Safety Authority, EFSA, as sufficiently characterized and identified at the strain level [10]) include inhibition of the growth of the food-borne human pathogen *Yersinia enterocolitica* [11] and anti-proliferative activity on both gastric and colon cancer cell lines [12]; more significantly, in vivo human trials demonstrated the effect of *L. paracasei* IMPC 2.1—associated with artichoke—on the modulation of fecal biochemical and microbiological parameters [13] and its efficacy in reducing symptoms of functional constipation [14], as also evaluated in a meta-analysis of randomized controlled trials on the effect of probiotics on gastrointestinal dysfunction [15]. The interaction with the immune system and its modulation are probably the most important mechanisms underlying the health-promoting effects of probiotic bacteria [16]. The immunomodulatory properties of strain IMPC 2.1 were evaluated in comparison to other strains of the same species, studying the effects of their interaction with dendritic cells (DCs), which are important in the earliest bacterial recognition and in determination of the subsequent T-cell responses, playing a pivotal role in both innate and adaptive immunity [16]. The results of the study [17] indicated that strain IMPC 2.1, as well as the other strains, stimulated phenotypic maturation of DCs. Moreover, the study confirmed that different strains—even of the same species—may show very diverse immunomodulatory properties. In fact, strain IMPC 2.1 induced a low pro-inflammatory response (enough to induce a state of alertness of the immune system), while strain IMPC 4.1 (genetically similar to IMPC 2.1 [10]) was characterized by very interesting and peculiar anti-inflammatory properties. Although a number of studies indicate that the immunomodulatory properties of probiotic bacterial strains are due to molecules associated to the bacterial cell surface [18,19], recent increasing evidence indicates that they can also be due to the production of specific compounds which are secreted in the culture medium. For example, Thomas et al. [20] demonstrated that the probiotic strain *Lactobacillus reuteri* 6475 secretes histamine, which inhibits the production of the pro-inflammatory cytokine tumor necrosis factor (TNF). In another study, *L. plantarum* 10hk2 was shown to produce a protein fraction estimated to have a molecular weight of 8.7 kDa with anti-inflammatory activity [21]. A proteinaceous compound with a molecular weight of 50 kDa is secreted in the culture supernatant of *Bifidobacterium animalis* subsp. *lactis* strain BB12, and it was found to be involved in the anti-inflammatory effects caused in stimulated Caco-2 cells by that bacterial strain [22]. Concerning *L. paracasei*, an anti-inflammatory activity was also detected in a culture supernatant of strain B21060, as it was able to inhibit the production of inflammatory cytokines by DCs in response to *Salmonella typhimurium* [23]. Likewise, a more recent work [24] highlighted that yet-unknown substances produced by a different *L. paracasei* strain were responsible for its anti-inflammatory properties, also indicating the possibility of exploiting this strain’s feature directly using a milk fermented with the strain. In those cases, *L. paracasei* compounds responsible for the anti-inflammatory activity remained unidentified, while von Schillde et al. [25] identified a lactocepin with a molecular weight higher than 100 kDa as a protease secreted by a strain of *L. paracasei*, exerting an anti-inflammatory activity by degrading pro-inflammatory chemokines. The above-mentioned studies indicated that the specific immunomodulatory activity of different probiotic strains can be due to diverse compounds whose production also depends on the composition of the culture medium. In this regard, it is important to note that the culture medium itself can also exert an immunomodulatory activity, thus hiding the results of in vitro experiments carried out to evaluate the immunomodulatory activity of bacterial culture filtrates [24]. In this context, the aim of this study is to further characterize the *L. paracasei* strains used in our previous work [17]—in particular strain IMPC 2.1—to ascertain if their immunomodulatory properties are related to metabolites released in the culture medium. Taking into account that the immunomodulatory activity of each strain is strictly strain-specific, and that this activity could be caused by diverse immunomodulatory compounds, the work carried out in this study represents an opportunity to highlight different immunomodulatory activities caused by novel molecules. Moreover, considering that several probiotic features of the strain *L. paracasei* IMPC 2.1 are exerted in the presence of the artichoke matrix, in order to reveal a potential symbiotic activity, we also evaluated the effect of an artichoke phenolic extract fermented by those *L. paracasei* strains on the immunomodulation of DCs. A further element of the novelty of this study is the development of an experimental system in which the bacterial strains were grown directly in media originally designed for the culture of DCs, thus avoiding the interfering effect of bacterial culture medium on DCs [24].

## 2. Materials and Methods

### 2.1. Artichoke Phenolic Extract Preparation and Assay

The edible part of fresh artichoke (cv. Violetto di Provenza) buds was used to prepare an artichoke phenolic extract (APE) in the form of a lyophilized powder, according to Di Venere et al. [9], with some modifications. Briefly, about 100 g of fresh tissue were extracted after homogenization (1:5 w/v) by refluxing in boiling methanol (twice for 1 h). The obtained extract was filtered through a Whatman 1 filter paper and then concentrated under reduced pressure by a rotary evaporator. The obtained dry residue was finally dissolved in 500 mL of distilled water. The resulting solution was filtered through a Whatman 1 filter paper, immediately frozen, and then lyophilized to obtain about 4.3 g of dry powder (APE), which was stored under vacuum at −25 °C until use. The phenolic content of the APE was assayed as follows. A weighed aliquot of APE was dissolved in a known volume of distilled water to realize a solution at concentration of about 4 mg/mL (w/v). The total phenolic concentration of the solution was quantified by high performance liquid chromatography (HPLC), as described below, and the total phenol (TP) content of APE was calculated as the sum of the contents of single peaks identified by HPLC analysis and expressed as mg of TP/g of dry powder.

### 2.2. Phenolic Composition and Concentration Assay

The analysis of phenolic compounds in artichoke extract, in the medium (used as a control), and in bacterial culture filtrates was performed by HPLC according to Gatto et al. [26]. Chromatographic peaks were identified according to Mileo et al. [27]. Calibration curves built for each compound using commercial standards were used to quantify the identified phenolics: 5-*O*-caffeoylquinic acid (5-*O*-CQA, also known as chlorogenic acid, CHLGA), 1-*O*-CQA, 3-*O*-CQA, 4-*O*-CQA, 1,3-dicaffeoylquinic acid (1,3-DCQA, also known as cynarin, CYN), 1,4-DCQA, 4,5-DCQA, 3,5-DCQA, 1,5-DCQA, 3,4-DCQA, and an apigenin glycoside (APG-GLYC), this latter being quantified as apigenin-7-glucoside.

### 2.3. Chemicals

HPLC grade water was obtained by a Milli-Q system (Millipore, Bedford, MA, USA). Methanol (Chromasolv^®^ gradient grade) was purchased from Sigma-Aldrich (Milan, Italy). All HPLC standards (chromatographic purity > 95%) were purchased from Phytolab GmbH and Co. KG (Vestenbergsgreuth, Germany).

### 2.4. Bacterial Strains and Culture Conditions

*L. paracasei* LMG 23554 (=strain YS8866441) was used as a certain non-probiotic strain [28], it was obtained from the Belgian Coordinated Collections of Microorganisms, Ghent, Belgium. *L. paracasei* IMPC 2.1 and IMPC 4.1 (both isolated from human intestine) were obtained from the Culture Collection at the Istituto di Microbiologia, Università Cattolica, Piacenza, Italy and deposited as strain LMG P-22043 and LMG S-27068 in the Belgian Coordinated Collections of Microorganisms, respectively. For long-term storage, 1 mL aliquots of fresh cultures with 20% Bacto glycerol (Difco, Detroit, MI, USA) were frozen at −80 °C in 2 mL sterile cryovials (Nalgene, Rochester, NY, USA). For preparation of working cultures, strains were 2% (v/v) inoculated in de Man Rogosa Sharpe (MRS) broth (Difco, Detroit, MI, USA), grown for 24 h at 37 °C under anaerobic conditions, and subcultured in the same medium twice before use in experiments. To obtain culture filtrates to be tested on DCs, strains were grown in RPMI 1640 medium (Sigma, St. Louis, MO, USA) with 10% fetal calf serum (Sigma, St. Louis, MO, USA), 1% l-glutamine (Sigma, St. Louis, MO, USA), and 1% non-essential amino acid (EuroClone, Milan, Italy) (complete RPMI 1640) or X-Vivo 15 medium (without Phenol red and Gentamicin, Lonza, Verviers, Belgium) with or without the addition of APE. A suitable amount of APE was dissolved in the culture media to realize a final TP concentration of about 500 mg/L (about 3.3 mg of APE/mL of medium), then the media were sterilized by filtration (0.22 μm) before strain inoculation. To this purpose, working cultures were harvested by centrifugation (9000× *g* for 10 min at 4 °C), washed twice with sterile saline solution (NaCl 0.85%, w/v) and re-suspended in the same volume of supplemented RPMI 1640 medium or X-Vivo 15 medium (with or without APE). These cultures were 2% (v/v) inoculated in 30 mL of the same media and incubated at 37 °C under anaerobic conditions. The experiment was carried out in triplicate. The growth of each strain was monitored by measuring the plate counts and the optical density (OD) value at 600 nm. When OD_600_ reached the value 0.6 (corresponding to a cell density of ca. 3 × 10^8^ cfu/mL), cell-free supernatants were obtained by centrifugation (9000× *g*, 4 °C, 10 min) and filtration through 0.22 μm filters (International PBI, Milan, Italy).

### 2.5. Mice

BALB-c mice were maintained under pathogen-free conditions at the animal facility of the Institute of Food Sciences (accreditation no. DM.161/99). Mice were used at the age of 6–12 weeks and were euthanized by inhalation of anaesthesia with isoflurane. All experiments with mice were performed in accordance with European Union Laws and guidelines. All animal studies were approved by the review committee of the Health Ministry, General Division of Animal Health and of Veterinary Medicine, and performed according to European regulations (EU Directive 2010/63/EU).

### 2.6. Isolation and Growth of Bone Marrow-Derived Dendritic Cells

Murine DCs were generated according to a previously published method [29]. In brief, bone marrow cells from the femurs and tibiae of mice were flushed and bone marrow cell aliquots (1 × 10^6^) were diluted in RPMI 1640 medium (Sigma, St. Louis, MO, USA) supplemented with antibiotics (penicillin 100 IU/mL; streptomycin 100 IU/mL) and 20 ng/mL granulocyte-macrophage colony-stimulating factor (GM-CSF) (culture medium) before being seeded in 12-well plates (Falcon, Heidelberg, Germany). On days 3, 5, and 7, 1 mL/well of cell surnatant was centrifuged and pellet re-suspended in freshly prepared culture medium. On day 9, cells were pre-incubated with bacterial filtrates. Cell viability was microscopically evaluated by dye-exclusion test using Nigrosin (1% solution), and >90% live cells were found in all experiments. Fluorescence-activated cell sorting (FACS) analysis revealed that cells resulted >75% CD11b^−^ CD11c^+^.

### 2.7. Microbial Filtrate Challenge

Immature DCs (iDCs) were incubated for 24 h in the presence of undiluted microbial filtrates in RPMI 1640 or X-Vivo 15 media with or without the addition of APE. Following incubation, cells were treated with 1 μg/mL lipopolysaccharide (LPS) for 6 h (LPS pulse) to induce the maturation of DCs and were cultured for an additional 24 h in RPMI 1640 or X-Vivo 15 media. Cells were collected for FACS analysis. Spent media were centrifuged at 10,000× *g* for 10 min to eliminate any residual cells and cell debris, and supernatants were stored at −80 °C.

### 2.8. Fluorescence-Activated Cell Sorting (FACS) Analysis

DCs were stained with phycoerythrin (PE)- or fluorescein isothiocyanate (FITC)-conjugated antibodies (Abs) (BioLegend, San Diego, CA, USA) against CD80 and CD86. Cell staining was analysed using a CyFlow Space flow cytometer (Partec, Munster, Germany) and FlowJo software (Tree Star Inc., Ashland, OR, USA). For each Ab, an isotype control of the appropriate isotype subclass was used.

### 2.9. Analysis of Cytokine Production

Supernatants from DCs cultures were analysed for IL-12, TNF-α, and IL-10 protein levels using an in-house sandwich ELISA. First, 100 μL aliquots of capture antibody solution (BioLegend, San Diego, CA, USA) were plated into ELISA wells (Nunc Maxisorb; eBioscience Inc., San Diego, CA, USA) and incubated overnight at 4 °C. After the removal of the capture antibody solution, 100 μL of blocking buffer (phosphate-buffered saline (PBS) supplemented with 1% bovine serum albumin (BSA)) were added to each well and incubated at room temperature for 2 h. Next, cytokine standards and samples diluted in blocking buffer supplemented with 0.05% Tween-20 were added to respective wells and incubated for 2 h at room temperature. At the end of the incubation, three washing steps with PBS supplemented with 0.05% Tween-20 were performed, and 100 μL aliquots of biotinylated antibody solution were added to the wells and incubated for 1.5 h at room temperature. After three washes, streptavidin–horseradish peroxidase conjugate solution (1:1500 dilution; BioLegend, San Diego, CA, USA) was then added to the wells and incubated for 1 h at room temperature. Finally, after washing, 100 μL of 63 mM Na_2_HPO_4_, 29 mM citric acid (pH 6.0) containing 0.66 mg/mL o-phenylenediamine/HCl and 0.05% hydrogen peroxide were dispensed into each well, and the wells were allowed to develop. The absorbance was read at 415 nm, and the cytokine concentrations were calculated using standard curves and expressed as pg/mL.

### 2.10. Statistical Analysis

Statistical significance was determined by *t*-test or ANOVA using GraphPad PRISM 4.0 software (GraphPad Software, Inc., La Jolla, CA, USA). A *p*-value of 0.05 or less was considered to be significant.

## 3. Results

### 3.1. Phenolic Content of Artichoke Extract and of Bacterial Culture Filtrates

**Figure 1:**
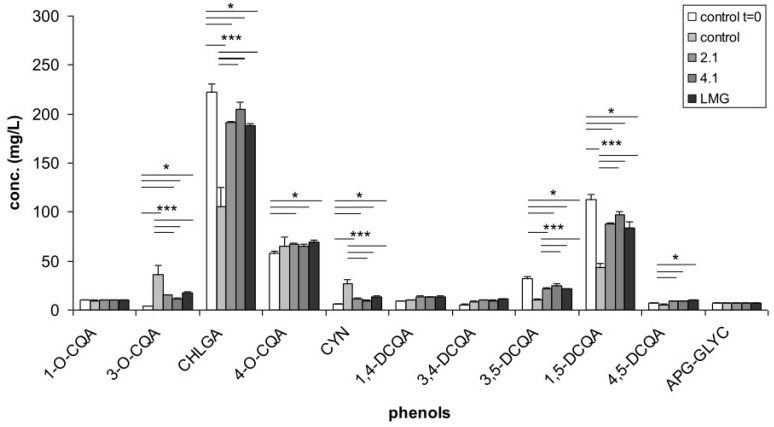
Concentration of phenolic compounds in the control (RPMI 1640 medium supplemented with artichoke phenolic extract) at the beginning (control *t* = 0) and at the end of incubation (control), as well as in bacterial culture filtrates after bacterial growth. Values represent the mean ± SD of three replications and statistical significance from ANOVA (* *p* < 0.05; ** *p* < 0.01; *** *p* < 0.001) is reported. 2.1: *Lactobacillus paracasei* IMPC 2.1; 4.1: *L. paracasei* IMPC 4.1; LMG: *L. paracasei* LMG 23554; CQA: Caffeoylquinic acid; CHLGA: Chlorogenic acid; CYN: Cynarin; DCQA: Dicaffeoylquinic acid; APG-GLYC: Apigenin glycoside.

The APE was found to contain CQAs, DCQAs, and small amounts of an APG-GLYC. The main components were CHLGA, 4-*O*-CQA, and two DCQAs (3,5-DCQA and 1,5-DCQA) (Figure 1). The total phenolic content of APE was found to be 151 mg TP/g dry powder. The concentration of the different phenolic compounds in complete RPMI 1640 supplemented with APE at the beginning (control *t* = 0) and at the end (control) of incubation, as well as in the bacterial culture filtrates after bacterial growth was determined (Figure 1). When APE was incubated in the presence of growing bacterial strains, a lower concentration of 3-*O*-CQA and CYN (about 50%) and a higher concentration of CHLGA, 3,5- and 1,5-DCQA (about 200%) were observed in comparison to the control (Figure 1). Such high differences in concentration were probably produced by phenolic structural changes occurring in the control during the incubation time, due to the slight alkaline pH of the culture broth (pH = 7.8). Actually, a slow isomerisation and breakdown of phenolic compounds at pH ≥ 7 have already been reported [30]. Therefore, the minimal changes in concentration of the different phenolic compounds observed in the three bacterial treatments might be explained by the progressive lowering of pH (down to pH = 6) produced by the bacterial growth in such samples during incubation, which probably contributed to an inhibition of the above-mentioned phenomena. No significant differences in phenolic composition was recorded among the three bacterial culture filtrates (Figure 1).

### 3.2. Growth of L. paracasei Strains in RPMI 1640 and X-Vivo 15 Media

Bacterial culture filtrates tested on DCs were obtained by growing bacterial strains in complete RPMI 1640 medium and X-Vivo 15. All three strains were similarly able to grow in complete RPMI 1640 medium, the presence of fetal calf serum being essential for growth, but notably also in X-Vivo 15, which it is a specific serum-free medium. The addition of APE to both media markedly improved the growth of the strains; in fact, an OD_600_ = 0.6 (corresponding to a cell density of ca. 3 × 10^8^ cfu/mL) was reached after about 24 h of incubation without APE and after 9 h with APE addition.

### 3.3. Effect of Culture Media on the Maturation of Dendritic Cells and Cytokine Production

**Figure 2:**
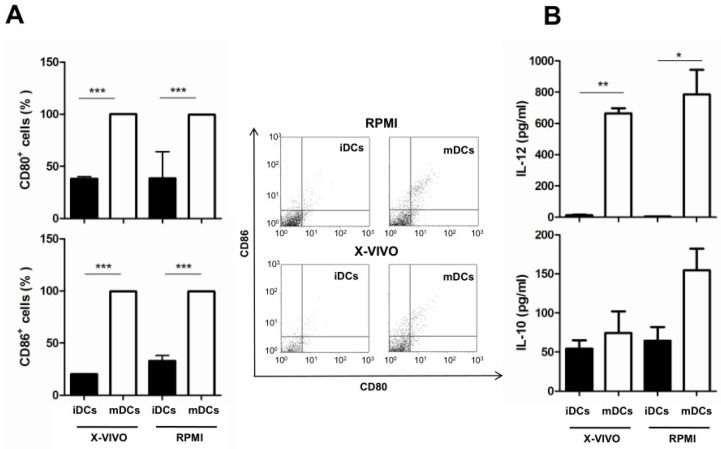
Effect of the culture medium RPMI 1640 (RPMI) or X-Vivo 15 (X-VIVO) on maturation of dendritic cells (DCs) and cytokine production. (**A**) Effect on maturation (induced by lipopolysaccharide (LPS) stimulation) revealed by the expression of CD86 and CD80 molecules; representative dot plots are also reported; (**B**) Effect on cytokine production. mDCs: Mature DCs; iDCs: Immature DCs; IL: Interleukin. The statistical significance (* *p* < 0.05; ** *p* < 0.01; *** *p* < 0.001) is reported.

Intestinal DCs are able to directly sample luminal antigens by extruding dendrites between epithelial cells. To reproduce this interaction in vitro, we pulsed bone marrow-derived immature DCs (iDCs) with LPS to obtain mature DCs (mDCs). Moreover, the effects of the two culture media were compared on both iDCs and mDCs (Figure 2). Following LPS-pulse, mDCs expressed significantly high levels of CD80 and CD86 co-stimulatory markers. This increase was comparable in both media (Figure 2A). Next, the effect of medium on the cytokine profile of DCs was analyzed by evaluating the production of interleukin IL-12 and IL-10, a pro- and an anti-inflammatory cytokine, respectively. As reported in Figure 2B, IL-12 significantly increased in mDCs, independently of the medium. On the contrary, RPMI 1640 favoured the expression of IL-10 in mDCs, even if levels were not found statistically relevant.

### 3.4. Effect of Bacterial Culture Filtrates with or without Artichoke Phenolic Extract on the Maturation of Dendritic Cells

**Figure 3:**
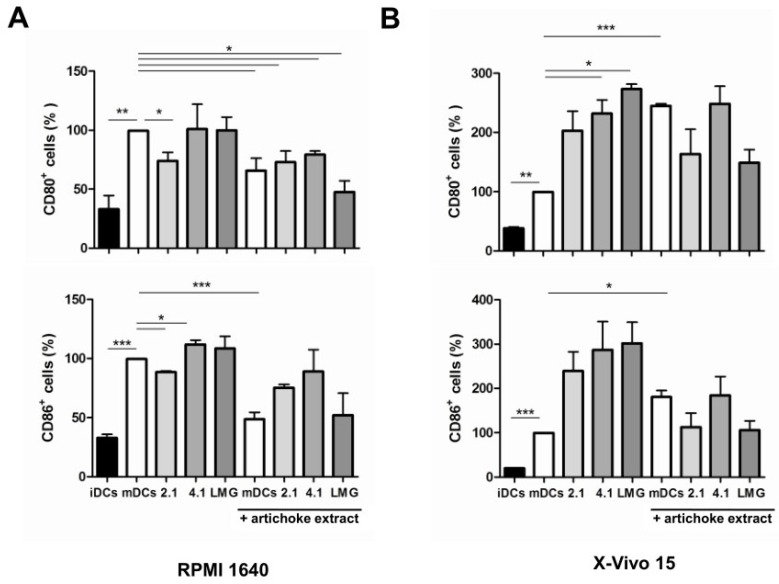
Effect of bacterial culture filtrates on the maturation of dendritic cells (DCs) as revealed by the expression of CD86 and CD80 molecules. (**A**) Effect caused by culture filtrates obtained after bacterial growth in RPMI 1640 medium without or with artichoke phenolic extract; (**B**) Effect caused by culture filtrates obtained after bacterial growth in X-Vivo 15 medium without or with artichoke phenolic extract. mDCs: Mature DCs (obtained by LPS treatment); iDCs: Immature DCs; 2.1: Bacterial strain *Lactobacillus paracasei* IMPC 2.1; 4.1: Bacterial strain *L. paracasei* IMPC 4.1; LMG: Bacterial strain *L. paracasei* LMG 23554. The statistical significance (* *p* < 0.05; ** *p* < 0.01; *** *p* < 0.001) is reported.

To evaluate the immunomodulatory potential of metabolites released in the culture media in the presence or absence of APE, iDCs were incubated for 24 h with filtrates derived from bacterial growth in complete RPMI 1640 and X-Vivo 15 media supplemented or not with APE. Following this treatment, iDCs were LPS-pulsed and cultured for an additional 24 h in the same respective medium. As reported above, LPS induced increased expression of both CD80 and CD86 surface markers in both examined media (Figure 3A,B). Pre-treatment of iDCs with the culture filtrate obtained after the growth of strain LMG 23554 in RPMI 1640 medium (Figure 3A) did not further stimulate their maturation, whereas the levels of CD80 and CD86 induced by IMPC 2.1 culture filtrate were significantly lower. On the contrary, IMPC 4.1 induced CD86 expression. In presence of APE-supplemented complete RPMI 1640 medium, LPS-induced CD80 and CD86 expressions were significantly reduced in mDCs. This inhibition was partially limited for CD86 by the bacterial fermentation of RPMI 1640 medium supplemented with APE (Figure 3A). Different effects on marker expressions were observed when DCs were pre-incubated with culture filtrates obtained after bacterial growth in X-Vivo 15 medium (Figure 3B). In fact, an increase of both DC markers was reported for all the three strains, although it was statistically significant only for CD80 expression observed after pre-incubation with IMPC 4.1 and LMG 23554. Additionally, the addition of APE to X-Vivo 15 medium caused a marked increase of both co-stimulatory markers. Pre-treatment of DCs with IMPC 2.1 and LMG 23554 filtrates obtained after growth in the presence of APE reduced this effect, even if this was not found to be statistically significant.

### 3.5. Effect of Bacterial Culture Filtrates with or without Artichoke Phenolic Extract on Cytokine Production by Dendritic Cells

**Figure 4:**
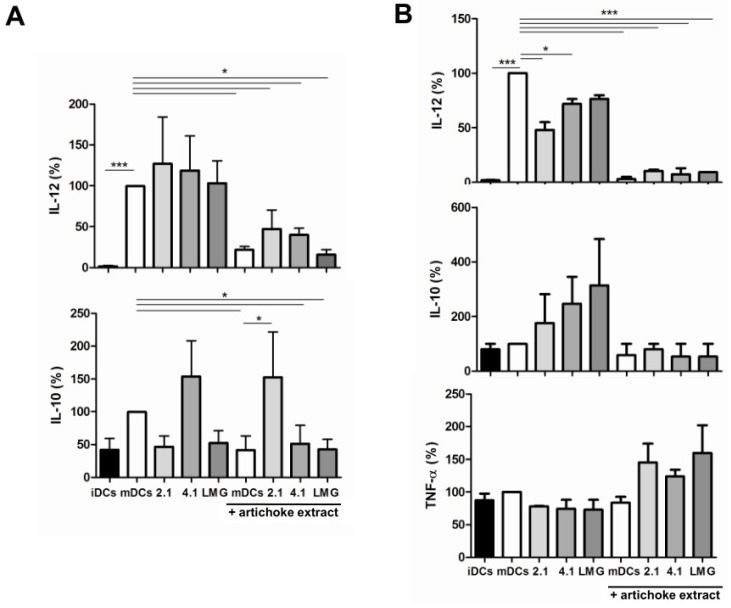
Effect of bacterial culture filtrates obtained after the growth in (**A**) RPMI 1640 medium or (**B**) X-Vivo 15 medium with or without artichoke phenolic extract on cytokine production by dendritic cells (DCs). mDCs: Mature DCs (obtained by LPS treatment); iDCs: Immature DCs; 2.1: Bacterial strain *Lactobacillus paracasei* IMPC 2.1; 4.1: Bacterial strain *L. paracasei* IMPC 4.1; LMG: Bacterial strain *L. paracasei* LMG 23554; TNF: Tumor necrosis factor; IL: Interleukin. The statistical significance (* *p* < 0.05; *** *p* < 0.001) is reported.

Next, the production of IL-12 and IL-10 by challenged DCs was determined. Treatment with culture filtrates obtained after the growth in RPMI 1640 medium did not modify IL-12 secretion of mDCs, but determined differential (albeit non-significant) effects on IL-10 production (Figure 4A). In particular, culture filtrates of strains IMPC 2.1 and LMG 23554 reduced the production of IL-10, whereas strain IMPC 4.1 increased the production of that cytokine. Notably, the addition of APE to the culture medium significantly reduced the production of both IL-12 and IL-10 by mDCs, with the exception of strain IMPC 2.1 for IL-10. In parallel, treatment of DCs with culture filtrates obtained after the growth of bacterial strains in X-Vivo 15 medium determined a decrease of IL-12 production; this effect was statistically significant for IMPC 2.1 and IMPC 4.1 strains (Figure 4B). On the contrary, culture filtrates tended to increase IL-10 production in mDCs. Notably, the addition of APE to X-Vivo 15 medium completely suppressed LPS-induced production of IL-12 and blocked IL-10 induction by filtrates. In this regard, it is interesting to note that the treatment of DCs with bacterial culture filtrates obtained after growth in X-Vivo 15 medium was cytokine-specific. In fact, a filtrate-dependent trend toward an increased production of TNF-α was observed in the presence of APE, even if it was not found to be statistically significant.

## 4. Discussion

In this study, *L. paracasei* strains have been further characterized to reveal whether their strain-specific immunomodulatory properties [17] could be related to the production of bioactive molecules in a culture medium. The most advanced trend in research on probiotic bacteria aims at the exploitation of these molecules [24] for medical applications. These compounds could allow more specific therapeutic effects to be obtained, even if their use would require complex studies to get the desired clinical results. Nevertheless, it should be considered that the identification of bioactive compounds responsible for a probiotic effect is very challenging, because the peculiar immunomodulatory properties of each bacterial strain could be the overall result of still-undefined factors, including a very complex mixture of bacterial metabolites [18,31]. Therefore, the use of a fermented milk—possibly containing a mixture of bioactive compounds—has been proposed to obtain an anti-inflammatory effect [24]. Moreover, the choice of the experimental conditions plays a fundamental role. Previous studies [24] demonstrated that a common medium (i.e., MRS) for the growth of lactobacilli itself has an effect on cytokine production by DCs, thus possibly hiding and interfering with the effect of bacterial metabolites. A result of our study is that both complete RPMI 1640 and the serum-free X-Vivo 15 were found to be suitable for bacterial growth, thus avoiding the above-mentioned problem and making the experimental conditions more appropriate for in vitro assessment of DC functionality. Notably, treatment of DCs with bacterial culture filtrates obtained after growth in X-Vivo 15 (but not in RPMI 1640) partially replicated the effects caused by the direct interaction with bacterial cells [17]. Specifically, increased expression of CD86 and of the anti-inflammatory cytokine IL-10 in mDCs were confirmed mainly for filtrates from X-Vivo 15. On the contrary, these filtrates decreased IL-12 levels in mDCs, rather than inducing, as reported following bacterial cell interaction [17]. Importantly, these data strongly suggest the existence of soluble metabolites that were functionally active (immunomodulatory) only in a serum-free medium. Further studies are then requested to assess if interaction with protein components or hydrolases can dampen these activities in complete RPMI 1640 medium. Nevertheless, interesting results were obtained in both media when an artichoke phenolic extract (APE) was added. It is interesting to note that APE addition did not inhibit bacterial growth, but improved it in both media. As the analysis of artichoke phenols did not reveal significant variations, this effect could be due to unidentified compound/s. Concerning the immunomodulatory effect, the addition of APE significantly reduced the expression of both IL-12 and IL-10 in mDCs. Among the examined filtrates, only that derived from the growth of strain IMPC 2.1 in RPMI 1640 was able to recover IL-10 production. It is well known that the association of a probiotic bacterium with the appropriate food matrix is very important for its efficacy and in determining the kind of health-promoting effect [32]. In this case, the addition of APE to the RPMI 1640 medium resulted in a culture filtrate obtained after the growth of strain IMPC 2.1 with interesting anti-inflammatory properties, as it increased the production of the anti-inflammatory cytokine IL-10 while decreasing IL-12 production. It is noteworthy that strain IMPC 2.1 is the *L. paracasei* strain used for the development of the innovative “probiotic artichoke” [4], and that the anti-inflammatory effect is strictly strain-specific, differentiating strain IMPC 2.1 from the other tested strains. Therefore, the anti-inflammatory effect is not due to the APE itself but to the interaction between APE and the strain metabolism. As the analysis of artichoke phenols in bacterial culture filtrates did not reveal significant differences between strains, it could be hypothesized that APE modified IMPC 2.1 metabolism, inducing the production of one or more still-unknown anti-inflammatory metabolites. Another plausible hypothesis is that APE did not modify strain metabolism, but that the anti-inflammatory effect is the result of a complex interaction between bacterial metabolites and APE compounds with DCs. Evaluation of these hypotheses could be the subject of future interesting studies. In conclusion, the results of this study indicate that the immunomodulatory properties of a probiotic strain may also depend on the particular medium used for its growth. Therefore, the choice of the right culture medium plays a fundamental role in research activities aimed at the production of immunomodulatory compounds. In this case, an interesting anti-inflammatory activity was detected in the culture filtrate obtained by growing a probiotic *L. paracasei* strain in a medium for DCs supplemented with APE. Therefore, the anti-inflammatory activity of a probiotic strain is synergistically joined with the antioxidant and the other health-promoting properties of artichoke phenolic compounds in that culture filtrate [27,33,34], paving the way to future interesting practical applications.
